# Complications of central venous catheterization at a vascular surgery service in a teaching hospital: a prospective cohort study

**DOI:** 10.1590/1677-5449.202300702

**Published:** 2023-09-18

**Authors:** Leonardo Jatczak, Renan Camargo Puton, Alencar Junior Lopes Proença, Leonardo Colussi Rubin, Luiza Brum Borges, Jaber Nashat Saleh, Mateus Picada Corrêa

**Affiliations:** 1 Faculdade Meridional - IMED, Passo Fundo, RS, Brasil.; 2 Instituto Vascular de Passo Fundo - INVASC, Passo Fundo, RS, Brasil.; 3 Hospital de Clínicas de Passo Fundo - HCPF, Passo Fundo, RS, Brasil.; 4 Universidade de Passo Fundo - UPF, Passo Fundo, RS, Brasil.

**Keywords:** vascular access devices, catheters, postoperative complications

## Abstract

**Background:**

Central venous catheters are essential for management of hospitalized patients, but their insertion is subject to complications that can make them unusable and/or cause patient morbidity. There are few data on the incidence of these complications and the variables associated with these outcomes in Brazil.

**Objectives:**

To determine the incidence of mechanical complications and failures of short stay central venous catheters fitted by the vascular surgery service at a teaching hospital and identify variables associated with their occurrence.

**Methods:**

This was a prospective cohort of 73 attempts to fit patients with a central venous catheter performed by the vascular surgery service at a teaching hospital from July to October of 2022.

**Results:**

Mechanical complications occurred in 12 cannulation attempts (16.44%) and there were 10 failures (13.70%). The factors associated with mechanical complications were less experienced operators (p < 0.001), less specialized operators (p = 0.014), a failed attempt prior to requesting help from the vascular surgery service (p = 0.008), and presence of at least two criteria for difficulty (p = 0.007).

**Conclusions:**

The local incidence of mechanical complications and central venous cannulation failures was similar to rates described in the international literature, but higher than rates in other Brazilian studies. The results suggest that the degree of experience of the person fitting the catheter, history of a failed prior attempt, and presence of at least two criteria for difficulty identified before the procedure were associated with worse outcomes.

## INTRODUCTION

Central venous catheters are an essential part of care of hospitalized patients, because they enable administration of vesicant drugs, provision of dialysis, and provision of parenteral nutrition.^1^ It is estimated that around 250,000 central venous catheters are fitted annually in the United Kingdom and the rate exceeds 5 million in the United States.^2,3^ In Brazil, although large scale epidemiological data are rare, the Unified Health System (SUS - Sistema Único de Saúde) authorized placement of approximately 95,000 central venous catheters in 2015, including both long and short stay devices.^4^

Problems related to the central venous cannulation procedure defined as mechanical complications include punctured arteries, hematoma, bleeding, incorrect positioning, pneumothorax, and nerve damage.^5,6^ The rate of mechanical complications is estimated at 5 to 19% of cannulation attempts, of which arterial puncture is the most common, occurring in around 4.2 to 9.3% of procedures.^7-9^

In view of the scarcity of published data on the mechanical complications of central venous cannulation in Brazil, the objective of this study was to analyze the rate of complications of placement of short stay central venous catheters performed by the vascular surgery service at the Hospital de Clínicas de Passo Fundo.

## METHODS

This is a longitudinal, observational, analytical, prospective cohort study. It was submitted to and approved by the Teaching and Research Administration at the Hospital de Clínicas de Passo Fundo, under protocol 461PPes, and the Faculdade Meridional Ethics Committee, under Ethics Appraisal Submission Certificate 58763522.6.0000.5319. All participants or their relatives were given a free and informed consent form, which was read and signed before any data were collected.

Patients were recruited who underwent attempts to fit a short stay central venous access (non-tunneled catheters) with direct assistance (execution of the procedure itself) or indirect assistance (help with definition of the most appropriate puncture site and technique) by the vascular surgery service at the Hospital de Clínicas de Passo Fundo from July 15, 2022 to October 15, 2022, over a total of 3 months of observation. Patients were excluded from the final sample if they refused to participate in the study or underwent the procedure in emergency scenarios (cardiorespiratory arrest or severe arrhythmia) or if it was not possible to obtain the free and informed consent form before performing the procedure.

The primary objective was to determine the incidence of mechanical complications during attempts to insert short stay central venous catheters. Secondary objectives were to determine the incidence of failed procedures and the number of skin punctures per cannulation attempt and to identify variables associated with these outcomes.

### Sample and procedures

**Figure 1:**
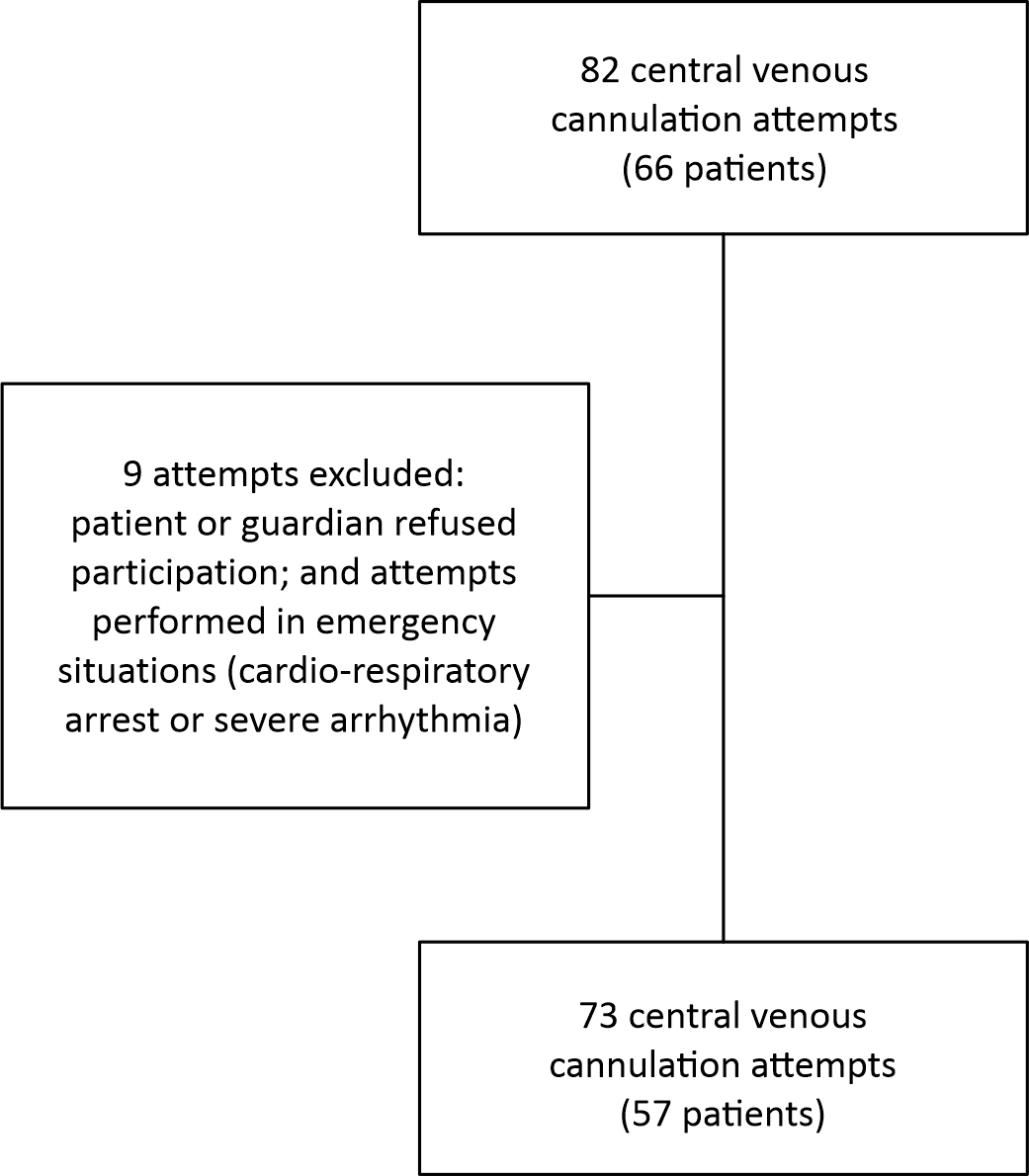
Flow diagram showing recruitment of cases and exclusions.

The initial sample, selected by convenience, comprised 82 central venous access attempts in a total of 66 patients. Nine attempts were excluded from this total (n = 73), as illustrated in Figure 1. With a view to verifying the reproducibility of the sample results, a post hoc sample size calculation was performed using G*Power (University of Düsseldorf, Germany), revealing a statistical test power of 0.80 with α = 0.05.

All procedures were performed using the Seldinger technique by health professionals who had graduated in medicine or by sixth-year medical students on obligatory placements as part of the curriculum and under direct supervision by a physician. Ultrasound guidance was used for all attempts to obtain access via the internal jugular, as per the standard protocol at the service, while use of ultrasound for subclavian and femoral accesses was according to operator preference.

Data on the procedures and their immediate outcomes were collected via direct observation by vascular surgery residents or another staff member (a physician or nursing technician), for cases of access attempts performed by the vascular surgery residents themselves. The researchers monitored patients daily for 48 hours after each attempt to screen for development of mechanical complications. Correct positioning of the catheter tips was confirmed by observation of control X-rays.

### Variables

Table 1 lists the main variables collected. Level of operator experience was dichotomized as greater than or equal to 50 central venous accesses placed in the operator’s entire academic-professional career or less than 50, in line with previous studies.^7,10,11^ Patient variables correlated with need for a higher number of skin punctures per access attempt and with higher incidence of mechanical complications, termed as “criteria for difficulty” in the literature,^12-15^ were also systematically screened before each attempt, as described in Table 2.

**Table 1:** Frequency of variables related to central venous cannulation attempts.

	*n = 73*
**Age (range), years**	66 (18-89)
**Genus/gender, n (%)**	
Female	37 (50.69)
Male	36 (49.31)
**Prior history of central venous cannulation, n (%)**	27 (36.99)
**Comorbidities, n (%)**	
Kidney disease	40 (54.79)
Chronic kidney disease	26 (35.62)
Acute kidney injury	21 (28.77)
Infection	24 (32.88)
Diabetes mellitus	19 (26.03)
Heart disease	19 (26.03)
Cancer	17 (23.29)
Lung disease	15 (20.55)
**Degree of specialization of the person performing cannulation, n (%)**	
General surgeon on 1st year of vascular surgery residency	45 (61.64)
First year resident on direct access residency (Internal Medicine, General Surgery, Neurosurgery)	17 (23.29)
Sixth year medical student	11 (15.07)
**Level of experience of the person performing cannulation, n (%)**	
≥ 50 central venous accesses	45 (61.64)
< 50 central venous accesses	28 (38.36)
**State of consciousness of patient during the procedure, n (%)**	
Alert	57 (78.08)
Altered state of consciousness	16 (21.92)
**Patient on invasive positive pressure ventilation, n (%)**	13 (17.80)
**Patient’s position during the procedure, n (%)**	
Horizontal supine	67 (91.78)
Trendelenburg position	6 (8.22)
**Class of procedure, n (%)**	
Elective	59 (80.82)
Urgent	14 (19.18)
**Setting of procedure, n (%)**	
Ward	21 (28.76)
Hemodialysis	16 (21.92)
Emergency	13 (17.81)
Surgical center	11 (15.07)
ICU	8 (10.96)
Cath lab	4 (5.48)
**Specialty of primary assisting physician, n (%)**	
Nephrology	29 (39.72)
Internal medicine	13 (17.81)
Vascular surgery	7 (9.59)
Hematology	5 (6.85)
Orthopedics	5 (6.85)
General Surgery	4 (5.48)
Critical care	3 (4.11)
Neurosurgery	3 (4.11)
Digestive surgery	2 (2.74)
Coloproctology	1 (1.37)
Endoscopy	1 (1.37)
**Reason for requesting assistance of vascular surgery service, n (%)**	
Unspecified (request from assisting physician)	45 (61.64)
Difficult access anticipated	11 (15.07)
Failure of prior attempt(s)	10 (13.70)
**Reason for central venous access, n (%)**	
Hemodialysis	32 (43.83)
Infusion of irritant and/or vesicant solutions	14 (19.18)
Rapid infusion of drugs and/or blood products	14 (19.18)
Difficulty obtaining peripheral venous access	9 (12.33)
Total parenteral nutrition	4 (5.48)
**Number of catheter lumens in attempt to obtain central venous access, n (%)**	
Two lumens	41 (56.17)
Three lumens	32 (43.83)
**Use of ultrasound during puncture, n (%)**	58 (79.45)
**Puncture site, n (%)**	
Right internal jugular vein	35 (47.94)
Left internal jugular vein	20 (27.40)
Right femoral vein	9 (12.33)
Left femoral vein	1 (1.37)
Right subclavian vein	7 (9.59)
Left subclavian vein	1 (1.37)

ICU = intensive care unit.

**Table 2:** Frequency of criteria for difficulty of central venous accesses.

**Presence of criteria for difficulty of central venous access, n (%)**	**n = 73**
	**51 (69.86)**
*Clinical criteria*	43 (58.90)
Obesity (BMI > 30 kg/m^2^)	21 (28.77)
Difficulty cooperating	18 (24.66)
Prior history of mechanical complications related to central venous cannulation	15 (20.55)
Hypotension (SBP < 90 mmHg)	13 (17.81)
Local swelling	8 (10.96)
Recent local surgery and/or radiotherapy	8 (10.96)
Thyromental distance < 60 mm	4 (5.48)
Local arterial pulse difficult to palpate	4 (5.48)
Low tolerance of supine position	2 (2.74)
Local injury	1 (1.37)
*Laboratory criteria*	12 (16.44)
Platelet count < 50,000 cells/mm^3^	7 (9.59)
INR > 1.8	7 (9.59)
Other coagulopathies	1 (1.37)
*Ultrasonographic criteria*	12 (16.44)
Small veins (< 5 mm in diameter)	6 (8.22)
Veins difficult to visualize	4 (5.48)
Non-distensible veins	3 (4.11)

INR = international normalized ratio; SBP = systolic blood pressure; BMI = body mass index.

### Outcomes

A “central venous cannulation attempt” was defined as any, successful or unsuccessful, attempt to insert a venous catheter into the internal jugular, subclavian, or femoral vein by a single operator at a single time. The primary outcome was defined as all complications related to the procedure that occurred within 48 hours of the attempt.

Complications included arterial puncture (flow of bright-red blood red and high pressure in the puncture needle), hematoma (visible blood collection beneath the skin at the puncture site), important bleeding without hematoma (bleeding at the puncture site that needed compression to staunch, without formation of hematoma), pneumothorax (air in the pleural space visible on the control X-ray), incorrect catheter tip position (proximal extremity of the catheter outside the superior or inferior vena cava, cavoatrial junction, or right atrium), and nerve damage (sensory and/or motor deficit observed after the procedure, related to a nerve close to the puncture site, and not explainable by a different cause).

The secondary outcomes were incidence of failures (attempts in which it was not possible to insert the central venous catheter) and the number of skin punctures (where a puncture is defined as insertion of the puncture needle through the skin, regardless of whether it enters a central vascular structure, followed by its complete removal).

### Biases

With the objective of reducing the impact of selection bias, care was taken to enroll the majority of patients who met the inclusion criteria by rapid administration of the free and informed consent form before procedures. Observer bias was minimized by assigning data collection to a different professional than the person performing the cannulation and with conceptual standardization of variables and outcomes, as described above.

### Statistical analysis

Categorical variables were expressed as absolute and relative frequencies, and continuous variables as means and standard deviations (SD), when symmetrically distributed, or in the form of median and minimum and maximum values, if asymmetrically distributed. Outcomes were compared with the Mann-Whitney U test for continuous variables and the chi-square or Fisher’s exact test (when expected frequencies were less than five) for categorical variables. The effect size on qualitative outcomes was expressed as relative risk (RR), with a 95% confidence interval (95%CI). In turn, quantitative outcomes were expressed as means and with the Hodges-Lehmann estimator (HLE). All analyses were conducted using JASP 0.16.4.0 statistical software (University of Amsterdam, Netherlands). Results with p values < 0.05 were considered statistically significant.

## RESULTS

A total of 73 central venous cannulation attempts were identified in 57 patients, after application of the exclusion criteria, in patients under the primary or secondary care of the vascular surgery service at the Hospital de Clínicas de Passo Fundo. Of these, 12 patients needed two or more access attempts. Median age was 66 years (range: 18-89 years) and 50.69% of the patients were female. The most common reason for catheter placement was a need for hemodialysis (43.83%). The most common cannulation site was the internal jugular vein (75.34%). Ultrasound was used in 79.4% of the attempts, including all attempts to cannulate the internal jugular vein and three of the 10 femoral attempts. Table 1 lists the clinical and technical characteristics of the attempts analyzed.

Just seven of the total of 73 cannulation attempts (9.59%) were performed on patients cared for primarily by the vascular surgery service. Vascular surgery residents (all with ≥ 50 cannulations over their medical careers) performed 45 cannulation attempts, and in the remaining 28 attempts they only provided indirect assistance to other physicians or students. The most common criteria for difficulty observed were obesity (28.77%), poor patient cooperation (24.66%), and prior history of mechanical complications in central venous accesses (20.55%), as shown in Table 2.

Mechanical complications occurred in 16.44% of procedures, of which hematoma (9.59%) and arterial puncture (8.22%) were the most common. Two or more complications were observed in five procedures. Table 3 lists the distribution of study outcome incidence rates. The most frequent variables associated with incidence of mechanical complications were cannulation attempted by an operator with limited experience (RR 7.84, 95%CI 2.05-19.04, p < 0.001) and less specialized operators (residents of other specialties vs. vascular surgery residents, RR 6.62, 95%CI 1.50-15.93, p = 0.014; students vs. vascular surgery residents, RR 9.74, 95%CI 2.04-20.40, p = 0.002).

**Table 3:** Incidence of mechanical complications, failures, and number of skin punctures of central venous cannulation attempts.

	*n = 73*
**Presence of mechanical complications, n (%)**	12 (16.44)
Hematoma	7 (9.59)
Arterial puncture	6 (8.22)
Significant bleeding without hematoma	3 (4.11)
Incorrect catheter tip position	1 (1.37)
Pneumothorax	0 (0.00)
Nerve damage	0 (0.00)
**Failures to obtain central venous access, n (%)**	10 (13.70)
**Number of skin punctures per attempted central venous cannulation (standard deviation)**	2.31 (± 1.23)

A history of failed access attempt made immediately prior to requesting assistance from the Vascular Surgery service (up to 12 hours previously [RR 4.50, 95%CI 1.69-7.31, p = 0.008]), and presence of two or more criteria for difficulty obtaining access (RR 5.43, 95%CI 1.42-12.63, p = 0.007) were also associated with mechanical complications. None of the criteria for difficulty were statistically significant correlated with occurrence of complications. Table 4 lists correlations between the characteristics of cannulation attempts and the incidence of complications and failures.

**Table 4:** Incidence of complications and failures according to patient and procedural variables.

	**Mechanical complications, n (%)**	**p**	**Failures to obtain central venous access, n (%)**	**p**
**Degree of specialization of the person performing cannulation**				
General surgeon on 1st year of vascular surgery residence (n = 45)	2 (4.44)	**0.01**	3 (6.67)	0.33
First year resident on direct access residency (n = 17)	5 (29.41)		3 (17.65)	
Sixth year medical student (n = 11)	5 (45.45)	**< 0.01**	4 (36.36)	**0.02**
General surgeon on 1st year of vascular surgery residency (n = 45)	2 (4.44)		3 (6.67)	
Sixth year medical student (n = 11)	5 (45.45)	0.44	4 (36.36)	0.38
First year resident on direct access residency (n = 17)	5 (29.41)		3 (17.65)	
**Level of experience of the person performing cannulation**				
≥ 50 lifetime central venous accesses (n = 45)	2 (4.44)	**< 0.001**	3 (6.67)	**0.03**
< 50 lifetime central venous accesses (n = 28)	10 (35.71)		7 (25.00)	
**Reason for requesting assistance of vascular surgery service** (vs. other reasons)				
Unspecified (n = 45)	6 (13.33)	0.52	5 (11.11)	0.49
Difficult access anticipated (n = 11)	1 (9.09)	0.68	0 (0.00)	0.34
Failure of prior attempt(s) (n = 10)	5 (50.00)	**0.008**	4 (40.00)	**0.026**
**Number of criteria for difficulty identified during the procedure**				
Less than or equal to 1 (n = 38)	2 (5.26)	**0.007**	2 (5.26)	**0.029**
Greater than or equal to 2 (n = 35)	10 (28.57)		8 (22.86)	

With regard to the secondary outcomes, failures to obtain access occurred in 13.70% of attempts, and the mean number of skin punctures was 2.31 punctures per cannulation attempt (SD ± 1.23). Presence of failures was associated with limited operator experience (RR 3.75, 95%CI 1.09-8.81, p = 0.038), less specialized operators (students vs. vascular surgery resident, RR 5.45, 95%CI 1.42-11.36, p = 0.022), failure of immediately preceding cannulation attempt (RR 4.20, 95%CI 1.34-7.31, p = 0.026), and presence of at least two criteria for difficulty (RR 4.34, 95%CI 1.04-11.43, p = 0.029).

The number of skin punctures per attempt increased with presence of two or more criteria for difficulty (means 2.85 vs. 1.81, U = 358.5, HLE 1, p < 0.001), prior history of mechanical complications of central cannulation (means 3.06 vs. 2.12, U = 245.5, HLE 1, p = 0.007), limited experience of cannulation operator (means 3.18 vs. 1.78, U = 1,024, HLE 1, p < 0.001), and less specialized operators (resident of other specialty vs. vascular surgery resident, means 3.00 vs. 1.78, U = 595, HLE 1, p < 0.001; student vs. vascular surgery resident, means 3.44 vs. 1.78, U = 429, HLE 2, p < 0.001).

**Figure 2:**
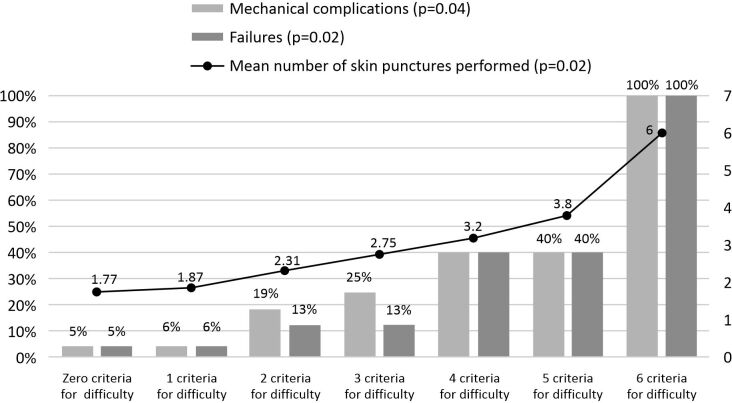
Incidence of outcomes by number of criteria for difficulty.

**Figure 3:**
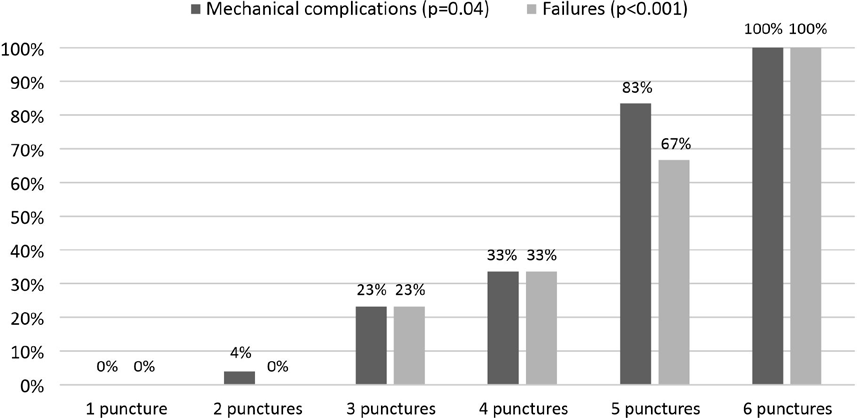
Incidence of complications and failures by number of skin punctures attempted.

The incidence of outcomes increased progressively with increasing number of criteria for difficulty and skin punctures, as illustrated in Figures 2 and 3, respectively. Mechanical complications were also more frequent when there were simultaneous cannulation procedure failures (RR 8.82, 95%CI 3.95-11.62, p < 0.001). Comparing the groups by different levels of experience with the procedure, it was found that this variable only remained associated with greater incidence of mechanical complications in the presence of at least two criteria for difficulty or of history of failed attempt within the previous 12 hours, since in the absence of both these variables, differences were not statistically significant, as shown in Table 5.

**Table 5:** Incidence of outcomes according to the experience of person performing procedure.

	**Mechanical complications, n (%)**	**p**	**Cannulation failure, n (%)**	**p**	**Mean number of skin punctures per access attempt (SD)**	**p**
**Presence of ≤ 1 criterion of difficulty(n = 38)**						
≥ 50 lifetime accesses (n = 24)	0 (0.00)	0.13	1 (4.17)	1.00	1.54 (± 0.83)	**0.003**
< 50 lifetime accesses (n = 14)	2 (14.28)		1 (7.14)		2.29 (± 0.73)	
**Presence of ≥ 2 criteria for difficulty (n = 35)**						
≥ 50 lifetime accesses (n = 21)	2 (9.52)	**0.006**	2 (9.52)	**0.04**	2.05 (± 0.74)	**< 0.001**
< 50 lifetime accesses (n = 14)	8 (57.14)		6 (42.86)		4.07 (± 1.14)	
**Absence of previous failed attempts (n = 63)**						
≥ 50 lifetime accesses (n = 40)	2 (5.00)	0.09	3 (7.50)	0.66	1.80 (± 0.85)	**< 0.001**
< 50 lifetime accesses (n = 24)	5 (21.73)		3 (12.50)		2.87 (± 1.21)	
**Presence of previous failed attempts (n = 10)**						
≥ 50 lifetime accesses (n = 5)	0 (0.00)	**0.008**	0 (0.00)	**0.048**	1.60 (± 0.55)	**0.01**
< 50 lifetime accesses (n = 5)	5 (100.00)		4 (80.00)		4.60 (± 0.54)	

SD = standard deviation.

## DISCUSSION

As far as is known, this is only the second prospective Brazilian study to investigate the outcome mechanical complications of central venous cannulation and is the first prospective study to be conducted in a teaching hospital. This cohort had a 16.44% incidence of mechanical complications of central venous cannulation and a 13.70% incidence of failures, compatible with the range of frequencies found in the international literature, which varies from 1.1 to 18% of procedures for complications and 0.4 to 22.3% for failures.^16-29^

In relation to Brazilian studies, the incidence of complications varies from 2.7 to 12% of procedures. Two of these three studies were observational and retrospective, performed in teaching hospitals with 1,502 and 311 central venous catheters inserted by medical residents, into internal jugular and subclavian sites, finding, respectively, mechanical complication rates of 2.7% and 6.5%.^18,19^ The third was a prospective observational study, with 421 hemodialysis catheters, the great majority (99%) inserted by angiologists or angiology residents, with a 12% incidence of mechanical complications.^30^ In turn, rates of failures, which were only reported in the second and third studies, were 3% and 2.4% of procedures respectively.

The greater occurrence of mechanical complications and failures observed in the present study may be because 15% of the procedures were performed by medicine interns, which was not the case in the other three studies, and also because of the presence of at least two criteria for difficulty in 47.94% of procedures, and failed prior attempts in 13.70% of attempts, both of which were factors associated with greater incidence of outcomes.

The factor most frequently associated in the literature with increased rates of mechanical complications and failures of central venous cannulation is a greater number of skin punctures, since three or more punctures increased the risk of mechanical complications by around six times.^7,12,16^ Other variables associated with increased incidence of complications include experience of the person performing cannulation, not using ultrasound during the procedure, and prior history of central venous cannulation.^5,6,10,31^ Patient and puncture site characteristics were also correlated with greater difficulty in obtaining and negative outcomes of central venous access.^13,14^

In the present study, procedures performed by caregivers with limited experience (< 50 central venous accesses) were related to greater numbers of mechanical complications, failures, and skin punctures per attempt, which have also been reported in several other studies.^5,10,16,17,21,22,32^ A history of failure of the immediately preceding cannulation attempt was also associated with higher rates of complications and with additional insertion failures, which has also been described before in the literature.^12,28,33^

Presence of factors related to greater difficulty inserting central venous catheters was associated with higher frequency of occurrence of mechanical complications and failures in some studies.^12,14^ Here, although these criteria for difficulty did not in isolation have significant correlations with the outcomes analyzed, when they were grouped together as two or more criteria, there was an increase in complications and failures directly proportional to the number of criteria, as illustrated in Figure 2.

As far as is known, this is the first study in the literature to correlate number of criteria for difficulty with incidence of complications and failures. Mechanical complications and failures also increased progressively with greater numbers of skin punctures per attempt, which was also observed in a series of other studies.^7,16,22,23,29,33,34^

In contrast with the majority of literature, this cohort did not exhibit significant differences in outcomes between cannulation attempts performed with or without ultrasound guidance. It should be pointed out that this study had asymmetrical proportions between groups with and without ultrasound guidance and also had operators who were fairly inexperienced, given that a training program is not yet available at this center for medical students or some of the direct access residencies. This result may suggest that use of ultrasound by untrained operators may not yield benefits for patients, and further studies, preferably multicenter, are needed to confirm or reject this hypothesis.

One interesting finding of this study was the fact that different degrees of experience and specialization only affected outcomes in the presence of failure of an immediately preceding attempt or at least two criteria for difficulty. This could be a consequence of the sample selected by convenience, but may also show that the lower rate of complications when cannulation is performed by experienced operators is restricted to more complex cases.

This study has several limitations. First, it is an observational study, i.e., it is not possible to rule out the hypothesis that the differences observed may be the result of omission of confounding variables. Second, although the sample was adequate for validation of the statistical tests, it was selected at a single center, because the researchers lacked the resources to conduct a multicenter study, and so the results should be interpreted with caution. Third, the great majority of the sample comprised cannulation attempts for which other specialties had requested assistance and cases with at least one criterion for difficulty, which could partially limit generalization of the results of this study. Fourth, not all cannulation attempts that met the inclusion criteria during the period were analyzed because it was not possible to administer the free and informed consent form or conduct data collection itself for some procedures, which were excluded from the study sample. Finally, there was a considerably greater proportion of cannulation attempts via jugular sites than via other sites.

Despite these limitations, this study adds to the literature the finding that only those central venous accesses that are theoretically more difficult may demand the greater expertise of a vascular surgeon or resident in the area to avoid complications. This may suggest that requests to the vascular surgery team for central venous access should be limited to difficult access, in order to avoid possibly limiting training of physicians and students with less experience with performing the procedure, in addition to sparing patients delays in provision of care.

## CONCLUSIONS

As far as it is known, this was the first prospective study in Brazil to assess the incidence of mechanical complications and failures of short-stay central venous cannulation at a teaching hospital. The incidence of outcomes was similar to rates described in the international literature, but greater than rates described in Brazilian studies. Predictive factors of greater frequency of complications and failures were the degree of experience and specialization of the person performing cannulation, a history of failure of an immediately prior attempt (up to 12 hours previously), and presence of at least two criteria for difficulty. However, there were no significant differences in outcomes between different levels of experience or different specialties in the absence of two other risk factors, suggesting that the benefit linked to performance of the procedure by a more experienced professional may be limited to difficult central venous accesses. Additional multicenter studies are needed to confirm the results of this study.
