# Supplementary material for: Complications of central venous catheterization at a vascular surgery service in a teaching hospital: a prospective cohort study
Source: J Vasc Bras. 2023 Sep 18;22:e20230070. doi: 10.1590/1677-5449.202300702 (PMC10545227; doi:10.1590/1677-5449.202300702)
Supplement: Translation

# Translation: Complicações de acessos venosos centrais em um serviço de cirurgia vascular de um hospital de ensino: uma coorte prospectiva

**DOI:** 10.1590/1677-5449.202300701

**Authors:** Leonardo Jatczak, Renan Camargo Puton, Alencar Junior Lopes Proença, Leonardo Colussi Rubin, Luiza Brum Borges, Jaber Nashat Saleh, Mateus Picada Corrêa

**Affiliations:** 1 Faculdade Meridional - IMED, Passo Fundo, RS, Brasil.; 2 Instituto Vascular de Passo Fundo - INVASC, Passo Fundo, RS, Brasil.; 3 Hospital de Clínicas de Passo Fundo - HCPF, Passo Fundo, RS, Brasil.; 4 Universidade de Passo Fundo - UPF, Passo Fundo, RS, Brasil.

**Keywords:** dispositivos de acesso vascular, cateteres, complicações pós-operatórias

## Abstract

**Contexto:**

Os acessos venosos centrais são essenciais no manejo de pacientes hospitalizados; contudo, a sua inserção está sujeita a complicações que podem dificultar seu uso e causar morbidade aos pacientes. No Brasil, dados acerca da incidência dessas complicações e das variáveis associadas com esse desfecho são escassos.

**Objetivos:**

Determinar a incidência de complicações mecânicas e de falhas de acessos venosos centrais de curta permanência realizados no serviço de Cirurgia Vascular de um hospital de ensino, além de identificar as variáveis associadas com a sua ocorrência.

**Métodos:**

Tratou-se de uma coorte prospectiva com 73 tentativas de acesso venoso central realizadas em pacientes assistidos pelo serviço de Cirurgia Vascular de um hospital de ensino entre julho e outubro de 2022.

**Resultados:**

Complicações mecânicas ocorreram em 12 tentativas de acessos (16,44%), e falhas, em 10 tentativas (13,70%). Os fatores associados às complicações mecânicas foram menor experiência do operador (p < 0,001), menor grau de especialização do operador (p = 0,014), falha na tentativa de acesso precedente à solicitação de auxílio ao serviço de Cirurgia Vascular (p = 0,008) e presença de pelo menos dois critérios de dificuldade (p = 0,007).

**Conclusões:**

A incidência local de complicações mecânicas e de falhas dos acessos venosos centrais foi semelhante à descrita na literatura internacional, mas foi superior à descrita em trabalhos brasileiros. Os resultados sugerem que o grau de experiência do executor do acesso, a história de falha em tentativa precedente e a presença de pelo menos dois critérios de dificuldade verificados antes do procedimento estão associados com piores desfechos.

## INTRODUÇÃO

Os acessos venosos centrais são fundamentais nos cuidados de pacientes hospitalizados, pois permitem a administração de drogas vesicantes, a terapia dialítica e o fornecimento da nutrição parenteral^1^ . Estima-se que cerca de 250.000 cateteres venosos centrais sejam inseridos anualmente no Reino Unido e que essa frequência seja de mais de 5 milhões nos Estados Unidos^2,3^ . No Brasil, embora dados epidemiológicos de grande escala sejam escassos, o Sistema Único de Saúde autorizou a inserção de aproximadamente 95.000 cateteres venosos centrais em 2015, incluindo dispositivos de curta e longa permanência^4^ .

As complicações denominadas mecânicas, relacionadas com o procedimento de cateterização venosa central, incluem punção arterial, hematoma, sangramento, posicionamento inadequado, pneumotórax e injúrias nervosas^5,6^ . A ocorrência de complicações mecânicas está estimada em 5 a 19% das tentativas de cateterização, sendo que punção arterial é a mais observada, ocorrendo em cerca de 4,2 a 9,3% dos procedimentos^7-9^ .

Diante da carência de dados na literatura nacional acerca das complicações mecânicas de acessos venosos centrais, esta pesquisa teve como objetivo analisar a taxa de complicações em acessos venosos centrais de curta permanência executados pelo serviço de Cirurgia Vascular do Hospital de Clínicas de Passo Fundo.

## MÉTODOS

Tratou-se de um estudo analítico, observacional e longitudinal, do tipo coorte prospectiva. O estudo foi submetido à avaliação e subsequentemente foi aprovado pela Coordenação de Ensino e Pesquisa do Hospital de Clínicas de Passo Fundo, sob o protocolo n.º 461PPes, e pelo Comitê de Ética da Faculdade Meridional, sob o Certificado de Apresentação de Apreciação Ética n.º 58763522.6.0000.5319. Todos os participantes ou seus familiares foram elucidados acerca do Termo de Consentimento Livre e Esclarecido (TCLE), com a leitura e a assinatura do documento prévias à coleta de quaisquer dados.

Foram incluídos pacientes submetidos a tentativas de acessos venosos centrais de curta permanência (cateteres não tunelizados) com assistência direta (execução do procedimento) ou indireta (auxílio na definição do sítio mais apropriado e na técnica de punção) do serviço de Cirurgia Vascular do Hospital de Clínicas de Passo Fundo, no período de 15 de julho de 2022 a 15 de outubro de 2022, totalizando 3 meses de atividade observacional. Foram excluídos da amostra final aqueles que recusaram a participação e aqueles submetidos ao procedimento em cenário de emergência (parada cardiorrespiratória ou arritmias graves), em que não foi possível a coleta do TCLE antes do procedimento.

O objetivo primário foi verificar a incidência de complicações mecânicas nas tentativas de inserção de cateteres venosos centrais de curta permanência. Os objetivos secundários foram determinar a incidência de falhas do procedimento e o número de punções da pele por tentativa de acesso, bem como identificar variáveis associadas com os desfechos.

### Amostra e procedimentos

A amostra inicial, obtida por conveniência, compreendeu 82 tentativas de acesso venoso central em um total de 66 pacientes, da qual foram excluídas nove tentativas de acesso venoso central (n = 73), conforme apresentado na Figura 1. Com vistas à verificação da reprodutibilidade dos resultados da amostra, foi realizado o cálculo amostral *post hoc*, através do *software* G*Power (Universidade de Düsseldorf, Alemanha), resultando em poder do teste estatístico de 0,80 na vigência de α = 0,05.

**Figura 1:** Fluxograma mostrando o recrutamento de casos e as exclusões.

Todos os procedimentos foram realizados com a técnica de Seldinger por profissionais graduados em Medicina ou por acadêmicos do sexto ano do curso de Medicina em estágio curricular obrigatório, mediante supervisão direta de um médico. O ultrassom foi usado para guiar todos os acessos tentados no sítio jugular interno, conforme normatização do serviço local; já nos sítios subclávio e femoral, seu uso esteve condicionado à preferência do operador.

Os dados referentes aos procedimentos e aos desfechos imediatos foram coletados através de observação direta pelos médicos-residentes de Cirurgia Vascular ou por outro colaborador (médico ou técnico de enfermagem), no caso dos acessos executados pelos próprios residentes em Cirurgia Vascular. Os pesquisadores acompanharam diariamente os pacientes por 48 horas após a tentativa para verificação do desenvolvimento de complicações mecânicas. A posição adequada da ponta dos cateteres inseridos foi confirmada através da observação das radiografias de controle.

### Variáveis

As principais variáveis coletadas estão citadas na Tabela 1. O nível de experiência do executor do acesso foi dicotomizado em maior ou igual e menor de 50 acessos venosos centrais inseridos com sucesso em toda a carreira acadêmico-profissional, em conformidade com estudos prévios^7,10,11^ . Variáveis do paciente correlacionadas com maior necessidade de punções da pele por tentativa de acesso e maior incidência de complicações mecânicas, conhecidas como “critérios de dificuldade” na literatura^12-15^ , também foram sistematicamente procuradas antes de cada tentativa, conforme descrito na Tabela 2.

**Tabela 1:** Frequência das variáveis nas tentativas de acesso venoso central.

	*n = 73*
**Idade (valor mínimo e valor máximo), anos**	66 (18-89)
**Gênero, n (%)**	
Feminino	37 (50,69)
Masculino	36 (49,31)
**História prévia de punção venosa central, n (%)**	27 (36,99)
**Comorbidades, n (%)**	
Doença renal	40 (54,79)
Doença renal crônica	26 (35,62)
Lesão renal aguda	21 (28,77)
Infecção	24 (32,88)
Diabetes melito	19 (26,03)
Doença cardíaca	19 (26,03)
Câncer	17 (23,29)
Doença pulmonar	15 (20,55)
**Grau de especialização do executor do acesso, n (%)**	
Médico-cirurgião geral do 1º ano de residência em Cirurgia Vascular	45 (61,64)
Médico do 1º ano de residência em residências de acesso direto (Clínica Médica, Cirurgia Geral, Neurocirurgia)	17 (23,29)
Acadêmico do 6º ano de Medicina	11 (15,07)
**Nível de experiência do executor do acesso, n (%)**	
≥ 50 acessos venosos centrais	45 (61,64)
< 50 acessos venosos centrais	28 (38,36)
**Estado de consciência do paciente durante o procedimento, n (%)**	
Lucidez	57 (78,08)
Alteração do estado de consciência	16 (21,92)
**Paciente sob ventilação invasiva com pressão positiva, n (%)**	13 (17,80)
**Posição do paciente durante o procedimento, n (%)**	
Decúbito dorsal plano	67 (91,78)
Posição de Trendelenburg	6 (8,22)
**Caráter do procedimento, n (%)**	
Eletivo	59 (80,82)
Urgente	14 (19,18)
**Local do procedimento, n (%)**	
Enfermaria	21 (28,76)
Hemodiálise	16 (21,92)
Emergência	13 (17,81)
Centro cirúrgico	11 (15,07)
UTI	8 (10,96)
Hemodinâmica	4 (5,48)
**Especialidade assistente primária do paciente, n (%)**	
Nefrologia	29 (39,72)
Clínica Médica	13 (17,81)
Cirurgia Vascular	7 (9,59)
Hematologia	5 (6,85)
Ortopedia	5 (6,85)
Cirurgia Geral	4 (5,48)
Medicina Intensiva	3 (4,11)
Neurocirurgia	3 (4,11)
Cirurgia Digestiva	2 (2,74)
Coloproctologia	1 (1,37)
Endoscopia	1 (1,37)
**Motivo da solicitação de assistência ao serviço de Cirurgia Vascular, n (%)**	
Não especificado (a pedido do médico assistente)	45 (61,64)
Acesso antecipado como difícil	11 (15,07)
Falhas em tentativas prévias de acesso	10 (13,70)
**Indicação do acesso venoso central, n (%)**	
Hemodiálise	32 (43,83)
Infusão de soluções vesicantes e/ou hiperosmolares	14 (19,18)
Rápida infusão de drogas e/ou hemocomponentes	14 (19,18)
Dificuldade na obtenção de acessos venosos periféricos	9 (12,33)
Nutrição parenteral total	4 (5,48)
**Número de lúmens do cateter usado na tentativa de estabelecimento do acesso venoso central, n (%)**	
Dois lúmens	41 (56,17)
Três lúmens	32 (43,83)
**Uso do ultrassom durante a punção, n (%)**	58 (79,45)
**Sítio de punção, n (%)**	
Veia jugular interna direita	35 (47,94)
Veia jugular interna esquerda	20 (27,40)
Veia femoral direita	9 (12,33)
Veia femoral esquerda	1 (1,37)
Veia subclávia direita	7 (9,59)
Veia subclávia esquerda	1 (1,37)

UTI = unidade de terapia intensiva.

**Tabela 2:** Frequência dos critérios de dificuldade à obtenção dos acessos venosos centrais.

Presença de critérios de dificuldade no estabelecimento do acesso venoso central, n (%)	n = 73
	51 (69,86)
Critérios clínicos	43 (58,90)
Obesidade (IMC > 30 kg/m^2^)	21 (28,77)
Difícil cooperação	18 (24,66)
História prévia de complicações mecânicas relacionadas à punção venosa central	15 (20,55)
Hipotensão (PAS < 90 mmHg)	13 (17,81)
Edema local	8 (10,96)
Cirurgia e/ou radioterapia local recente	8 (10,96)
Distância tireomentoniana < 60 mm	4 (5,48)
Pulso arterial local de difícil palpação	4 (5,48)
Baixa tolerância ao decúbito dorsal	2 (2,74)
Lesão local	1 (1,37)
Critérios laboratoriais	12 (16,44)
Contagem de plaquetas < 50.000 células/mm^3^	7 (9,59)
INR > 1,8	7 (9,59)
Outras coagulopatias	1 (1,37)
Critérios ultrassonográficos	12 (16,44)
Veias pequenas (< 5 mm de diâmetro)	6 (8,22)
Veias de difícil visualização	4 (5,48)
Veias não distensíveis	3 (4,11)

INR = razão normatizada internacional; PAS = pressão arterial sistólica; IMC = índice de massa corporal.

### Desfechos

A “tentativa de acesso venoso central” foi definida como a tentativa, com ou sem êxito, de inserção de um cateter venoso na veia jugular interna, subclávia ou femoral por apenas um operador em um único momento. O desfecho primário foi definido como todas as complicações relacionadas com o procedimento que ocorreram em um período de 48 horas da sua realização.

As complicações incluíram punção arterial (fluxo de sangue vermelho-vivo e de alta pressão pela agulha de punção), hematoma (coleção de sangue visível sob a pele do sítio puncionado), sangramento importante sem hematoma (sangramento no sítio de punção que exigiu compressão para sua cessação, sem a formação de hematoma), pneumotórax (presença de ar no espaço pleural visível na radiografia de controle), posição inadequada da ponta do cateter (extremidade proximal do cateter situada fora da veia cava superior ou inferior, junção cavo-atrial ou átrio direito) e injúria nervosa (déficit sensorial e/ou motor encontrado após o procedimento, referente a estrutura nervosa próxima do sítio de punção, não explicado por outra causa).

Os desfechos secundários compreenderam a incidência de falhas (tentativas em que não foi possível introduzir o cateter venoso central) e o número de punções da pele (sendo uma punção definida como a inserção de uma agulha de punção através da pele, atingindo ou não uma estrutura vascular central, seguida de sua retirada completa).

### Vieses

Com o intuito de reduzir o impacto do viés de seleção, procurou-se incluir a maioria dos pacientes que satisfizesse os critérios de inclusão através da rápida coleta do TCLE antes do procedimento. O viés do observador foi minimizado com a coleta dos dados por outro profissional que não o próprio executor do acesso e com a padronização conceitual das variáveis e desfechos como descrito anteriormente.

### Análise estatística

As variáveis categóricas foram descritas em frequências absolutas e relativas, e as contínuas, na forma de média e desvio-padrão (DP), quando de distribuição simétrica, ou na forma de mediana e valor mínimo e máximo, se distribuição assimétrica. Para a comparação dos desfechos entre variáveis, foram utilizados o teste U de Mann-Whitney para variáveis contínuas e os testes qui-quadrado ou exato de Fisher (quando ocorreram frequências esperadas menores de cinco) para as variáveis categóricas. O tamanho do efeito sobre os desfechos qualitativos foi representado na forma de risco relativo (RR), com intervalo de confiança de 95% (IC95%). Por sua vez, o desfecho quantitativo foi expresso através das médias e pelo estimador de Hodges-Lehmann (HLE). Todas as análises foram realizadas através do *software* estatístico JASP 0.16.4.0 (Universidade de Amsterdam, Países Baixos). Foram considerados como estatisticamente significativos valores de p < 0,05.

## RESULTADOS

Um total de 73 tentativas de acesso venoso central, em 57 pacientes, foi identificado após aplicação dos critérios de exclusão em pacientes assistidos, primária ou secundariamente, pelo serviço de Cirurgia Vascular do Hospital de Clínicas de Passo Fundo, sendo que 12 pacientes necessitaram de duas ou mais tentativas de acesso. A mediana de idade foi de 66 anos (18-89 anos), e 50,69% dos pacientes eram do sexo feminino. A principal indicação de inserção do cateter foi a necessidade de hemodiálise (43,83%). O sítio mais puncionado foi a veia jugular interna (75,34%). O ultrassom foi usado em 79,4% das tentativas, incluindo todas as tentativas empregadas na veia jugular interna e três das 10 tentativas no sítio femoral. A Tabela 1 apresenta as características clínicas e técnicas observadas nas tentativas analisadas.

De todas as 73 tentativas de acesso, apenas sete (9,59%) foram empregadas em pacientes assistidos primariamente pelo serviço de Cirurgia Vascular. Os médicos-residentes em Cirurgia Vascular (todos com ≥ 50 acessos na carreira médica) executaram 45 tentativas de acesso, sendo que, nas 28 restantes, apenas prestaram assistência indireta para outros médicos ou acadêmicos. Entre os critérios de dificuldade, os mais frequentemente encontrados foram obesidade (28,77%), difícil cooperação do paciente (24,66%) e história prévia de complicações mecânicas de acessos venosos centrais (20,55%), como exposto na Tabela 2.

Complicações mecânicas ocorreram em 16,44% dos procedimentos, sendo hematoma (9,59%) e punção arterial (8,22%) as mais comuns. Duas ou mais complicações foram registradas em cinco procedimentos. A Tabela 3 mostra a distribuição de incidências dos desfechos do estudo. Entre as variáveis associadas com a incidência de complicações mecânicas, destacaram-se a execução do acesso por um operador com experiência limitada (RR 7,84, IC95% 2,05-19,04, p < 0,001) e menor grau de especialização do operador (médico-residente em outras especialidades vs. residente em Cirurgia Vascular, RR 6,62, IC95% 1,50-15,93, p = 0,014; acadêmico vs. residente em Cirurgia Vascular, RR 9,74, IC95% 2,04-20,40, p = 0,002).

**Tabela 3:** Incidência de complicações mecânicas, falhas e número de punções da pele das tentativas de acesso venoso central.

	*n = 73*
**Presença de complicações mecânicas, n (%)**	12 (16,44)
Hematoma	7 (9,59)
Punção arterial	6 (8,22)
Sangramento importante sem hematoma	3 (4,11)
Posição inadequada da ponta do cateter	1 (1,37)
Pneumotórax	0 (0,00)
Injúria nervosa	0 (0,00)
**Falhas de aquisição do acesso venoso central, n (%)**	10 (13,70)
**Número de punções da pele por tentativa de estabelecimento do acesso venoso central (desvio-padrão)**	2,31 (± 1,23)

A história de falha em tentativa de acesso imediatamente anterior (até 12 horas antecedentes) à solicitação de assistência ao serviço de Cirurgia Vascular (RR 4,50, IC95% 1,69-7,31, p = 0,008) e a presença de dois ou mais critérios de dificuldade à obtenção do acesso (RR 5,43, IC95% 1,42-12,63, p = 0,007) também estiveram associadas com complicações mecânicas. Nenhum critério de dificuldade isolado apresentou correlação estatisticamente significativa com a ocorrência de complicações. A Tabela 4 correlaciona as características das tentativas de acesso com a incidência de complicações e falhas.

**Tabela 4:** Incidência de complicações e falhas de acordo com as variáveis dos pacientes e dos procedimentos.

	**Complicações mecânicas, n (%)**	**Valor de p**	**Falhas na obtenção do acesso venoso central, n (%)**	**Valor de p**
**Grau de especialização do executor do acesso**				
Médico-cirurgião geral do 1º ano de residência em Cirurgia Vascular (n = 45)	2 (4,44)	**0,01**	3 (6,67)	0,33
Médico do 1º ano de residência em residências de acesso direto (n = 17)	5 (29,41)		3 (17,65)	
Acadêmico do 6º ano de Medicina (n = 11)	5 (45,45)	**< 0,01**	4 (36,36)	**0,02**
Médico-cirurgião geral do 1º ano de residência em Cirurgia Vascular (n = 45)	2 (4,44)		3 (6,67)	
Acadêmico do 6º ano de Medicina (n = 11)	5 (45,45)	0,44	4 (36,36)	0,38
Médico do 1º ano de residência em residências de acesso direto (n = 17)	5 (29,41)		3 (17,65)	
**Nível de experiência do executor do acesso**				
≥ 50 acessos venosos centrais (n = 45)	2 (4,44)	**< 0,001**	3 (6,67)	**0,03**
< 50 acessos venosos centrais na vida (n = 28)	10 (35,71)		7 (25,00)	
**Motivo da solicitação de assistência ao serviço de Cirurgia Vascular** (vs. demais motivos)				
Não especificado (n = 45)	6 (13,33)	0,52	5 (11,11)	0,49
Acesso antecipado como difícil (n = 11)	1 (9,09)	0,68	0 (0,00)	0,34
Falhas em tentativas prévias de acesso (n = 10)	5 (50,00)	**0,008**	4 (40,00)	**0,026**
**Número de critérios de dificuldade encontrados durante o procedimento**				
Menor ou igual a 1 (n = 38)	2 (5,26)	**0,007**	2 (5,26)	**0,029**
Maior ou igual a 2 (n = 35)	10 (28,57)		8 (22,86)	

Entre os desfechos secundários, falhas de execução ocorreram em 13,70% das tentativas, e a média de punções da pele foi de 2,31 punções por tentativa de acesso (DP ± 1,23). A presença de falhas esteve associada com experiência limitada do operador (RR 3,75, IC95% 1,09-8,81, p = 0,038), menor grau de especialização do operador (acadêmico vs. residente em Cirurgia Vascular, RR 5,45, IC95% 1,42-11,36, p = 0,022), falha em tentativa de acesso imediatamente anterior (RR 4,20, IC95% 1,34-7,31, p = 0,026) e presença de pelo menos dois critérios de dificuldade (RR 4,34, IC95% 1,04-11,43, p = 0,029).

O número de punções da pele por tentativa aumentou na presença de dois ou mais critérios de dificuldade (médias 2,85 vs. 1,81, U = 358,5, HLE 1, p < 0,001), história prévia de complicações mecânicas de punções centrais (médias 3,06 vs. 2,12, U = 245,5, HLE 1, p = 0,007), menor experiência do executor do acesso (médias 3,18 vs. 1,78, U = 1.024, HLE 1, p < 0,001) e menor grau de especialização do operador (residente de outra área vs. residente de Cirurgia Vascular, médias 3,00 vs. 1,78, U = 595, HLE 1, p < 0,001; acadêmico vs. residente de Cirurgia Vascular, médias 3,44 vs. 1,78, U = 429, HLE 2, p < 0,001).

A incidência dos desfechos cresceu progressivamente com o aumento do número de critérios de dificuldade e de punções da pele, como representado nas Figuras 2 e 3, respectivamente. As complicações mecânicas também foram maiores na ocorrência simultânea de falhas do procedimento (RR 8,82, IC95% 3,95-11,62, p < 0,001). Comparando os grupos de diferente experiência no procedimento, evidenciou-se que essa variável se manteve associada com maior incidência de complicações mecânicas apenas na presença de pelo menos dois critérios de dificuldade ou da história de falha em uma tentativa nas 12 horas anteriores, de modo que, na ausência dessas variáveis, as diferenças não foram estatisticamente significativas, conforme apresentado na Tabela 5.

**Figura 2:** Incidência dos desfechos conforme o número de critérios de dificuldade.

**Figura 3:** Incidência de complicações e falhas conforme o número de punções da pele realizadas.

**Tabela 5:** Incidência de desfechos de acordo com a experiência do executor do procedimento.

	**Complicações mecânicas, n (%)**	**Valor de p**	**Falhas de acesso, n (%)**	**Valor de p**	**Média de punções da pele por tentativa de acesso (DP)**	**Valor de p**
**Presença de ≤ 1 critério de dificuldade (n = 38)**						
≥ 50 acessos na vida (n = 24)	0 (0,00)	0,13	1 (4,17)	1,00	1,54 (± 0,83)	**0,003**
< 50 acessos na vida (n = 14)	2 (14,28)		1 (7,14)		2,29 (± 0,73)	
**Presença de ≥ 2 critérios de dificuldade (n = 35)**						
≥ 50 acessos na vida (n = 21)	2 (9,52)	**0,006**	2 (9,52)	**0,04**	2,05 (± 0,74)	**< 0,001**
< 50 acessos na vida (n = 14)	8 (57,14)		6 (42,86)		4,07 (± 1,14)	
**Ausência de falhas em tentativas prévias (n = 63)**						
≥ 50 acessos na vida (n = 40)	2 (5,00)	0,09	3 (7,50)	0,66	1,80 (± 0,85)	**< 0,001**
< 50 acessos na vida (n = 24)	5 (21,73)		3 (12,50)		2,87 (± 1,21)	
**Presença de falhas em tentativas prévias (n = 10)**						
≥ 50 acessos na vida (n = 5)	0 (0,00)	**0,008**	0 (0,00)	**0,048**	1,60 (± 0,55)	**0,01**
< 50 acessos na vida (n = 5)	5 (100,00)		4 (80,00)		4,60 (± 0,54)	

DP = desvio-padrão.

## DISCUSSÃO

Pelo que se tem conhecimento, este é apenas o segundo estudo prospectivo brasileiro a avaliar o desfecho de complicações mecânicas de acessos venosos centrais, sendo o primeiro estudo prospectivo a ser conduzido em um hospital de ensino. Esta coorte apresentou uma incidência de complicações mecânicas de acessos venosos centrais de 16,44% e de falhas de 13,70%, compatíveis com a faixa de frequência encontrada na literatura internacional, que varia de 1,1 a 18% dos procedimentos para as complicações e de 0,4 a 22,3% para as falhas^16-29^ .

Em relação aos estudos nacionais, a incidência de complicações variou de 2,7 a 12% dos procedimentos. Dois dos três estudos foram observacionais e retrospectivos, realizados em hospitais de ensino com 1.502 e 311 cateteres venosos centrais inseridos por médicos-residentes, nos sítios jugular interno e subclávio, e que encontraram, respectivamente, taxas de complicações mecânicas de 2,7% e de 6,5%^18,19^ . O terceiro foi um estudo observacional prospectivo, com 421 cateteres de hemodiálise inseridos em sua grande maioria (99%) por angiologistas ou médicos-residentes em Angiologia, com uma incidência de 12% de complicações mecânicas^30^ . Por sua vez, as taxas de falhas, descritas apenas no segundo e no terceiro estudos, foram, respectivamente, de 3% e 2,4% dos procedimentos.

A maior ocorrência de complicações mecânicas e falhas no presente estudo pode ser explicada pela realização de 15% dos procedimentos por internos de Medicina, ausentes nos três outros estudos, bem como pela presença de pelo menos dois critérios de dificuldade em 47,94% dos procedimentos e de falhas em tentativas prévias de acesso em 13,70% das tentativas, ambos os quais foram associados com maior incidência dos desfechos.

O fator mais frequentemente associado com maiores taxas de complicações mecânicas e falhas de acessos venosos centrais na literatura é o maior número de punções da pele, visto que três ou mais punções aumentam em cerca de seis vezes o risco de complicações mecânicas^7,12,16^ . Outras variáveis associadas com o aumento da incidência de complicações são a experiência do executor do acesso, a ausência do ultrassom durante o procedimento e a história prévia de punções venosas centrais^5,6,10,31^ . Particularidades do paciente e do sítio de punção também foram correlacionadas com maior dificuldade e desfechos negativos dos acessos venosos centrais^13,14^ .

No presente estudo, os procedimentos realizados por prestadores com menor experiência (< 50 acessos venosos centrais) estiveram relacionados com mais complicações mecânicas, falhas e punções da pele por tentativa, o que também se refletiu em vários outros estudos^5,10,16,17,21,22,32^ . A história de falha em uma tentativa imediatamente anterior de acesso também associou-se com maiores taxas de complicações e com novas falhas de inserção, o que igualmente foi descrito na literatura^12,28,33^ .

A presença de fatores relacionados à maior dificuldade de inserção de cateteres venosos centrais foi associada à maior ocorrência de complicações mecânicas e de falhas em alguns estudos^12,14^ . Aqui, apesar de esses critérios de dificuldade não terem apresentado isoladamente correlação significativa com os desfechos analisados, quando estes estiveram associados em grupos de dois ou mais critérios, houve aumento das complicações e das falhas de modo diretamente proporcional ao número de critérios, como exposto na Figura 2.

Pelo que se tem conhecimento, este é o primeiro estudo na literatura a correlacionar o número de critérios de dificuldade com a incidência de complicações e falhas. As complicações mecânicas e as falhas também aumentaram progressivamente com o maior número de punções da pele por tentativa, o que também foi observado em uma série de outros trabalhos^7,16,22,23,29,33,34^ .

Diferentemente da maioria da literatura, esta coorte não demonstrou diferenças significativas nos desfechos entre as tentativas de acesso realizadas com ou sem o apoio do ultrassom. Cabe ressaltar que este estudo teve proporções assimétricas entre os grupos com e sem o uso do ultrassom e que também incluiu operadores um tanto inexperientes, dado que a presença de um programa de treinamento para acadêmicos e para algumas residências de acesso direto ainda não é uma realidade local. Tal resultado pode sugerir que o uso do ultrassom por operadores não treinados possa não adicionar benefícios ao paciente, sendo necessários novos estudos, de preferência multicêntricos, para confirmar ou rejeitar essa hipótese.

Um achado interessante deste estudo foi o fato dos graus de experiência e de especialização influenciarem os desfechos apenas na presença de falha em uma tentativa imediatamente precedente de acesso ou de pelo menos dois critérios de dificuldade. Isso pode ser uma consequência da amostra obtida por conveniência, mas pode também denotar que a menor taxa de complicações em acessos desempenhados por operadores experientes restringe-se a acessos de maior complexidade.

Este estudo possui várias limitações. Primeiro, é um estudo observacional, ou seja, não se pode excluir a hipótese de que as diferenças encontradas podem ser resultado da omissão de variáveis de confusão. Segundo, embora adequada para a validação dos testes estatísticos, a amostra pertence a um único centro, em razão da carência de recursos dos pesquisadores para realizar um estudo multicêntrico; por isso, os achados devem ser interpretados cautelosamente. Terceiro, a amostra foi constituída em sua grande maioria por acessos com solicitações de assistência por outras especialidades e com pelo menos um critério de dificuldade, o que pode limitar parcialmente a generalização dos resultados deste estudo. Quarto, nem todos os acessos que satisfaziam os critérios de inclusão no período avaliado foram abrangidos nesse trabalho, visto que, em alguns procedimentos, não foi possível a coleta do TCLE ou a própria coleta de dados, sendo excluídos, portanto, da amostra desta pesquisa. Por fim, a representatividade do sítio jugular foi consideravelmente maior que a dos demais sítios.

Apesar das limitações, o presente estudo acrescenta à literatura a constatação de que apenas os acessos venosos centrais teoricamente mais difíceis possam demandar da maior expertise de um cirurgião vascular ou médico-residente na área a fim de evitar complicações. Isso pode sugerir que a solicitação de acessos centrais para equipes de Cirurgia Vascular deve se ater a acessos difíceis, a fim de evitar uma possível limitação no treinamento de médicos e de acadêmicos com pouca experiência na execução do procedimento, além de poupar os pacientes de um atraso na prestação dos serviços assistenciais.

## CONCLUSÃO

Este foi o primeiro estudo prospectivo no Brasil, pelo que se tem conhecimento, a avaliar a incidência de complicações mecânicas e de falhas de acessos venosos centrais de curta permanência em um hospital de ensino. A incidência dos desfechos foi semelhante à descrita na literatura internacional, mas foi superior à descrita nos trabalhos brasileiros. Os preditores da maior ocorrência de complicações e falhas foram o grau de experiência e de especialização do executor do acesso, a história de falha em tentativa imediatamente precedente (até 12 horas anteriores) e a presença de pelo menos dois critérios de dificuldade. Contudo, não houve diferenças significativas de desfechos entre diferentes graus de experiência e de especialização do executor na ausência dos outros dois fatores de risco supracitados, sugerindo que o benefício atrelado à execução do procedimento por um profissional mais experiente pode limitar-se a acessos venosos centrais difíceis. Novos estudos multicêntricos são necessários para confirmar os resultados desta pesquisa.
